# Strain Dependent Variation of Immune Responses to *A. fumigatus*: Definition of Pathogenic Species

**DOI:** 10.1371/journal.pone.0056651

**Published:** 2013-02-18

**Authors:** Lisa Rizzetto, Gloria Giovannini, Michael Bromley, Paul Bowyer, Luigina Romani, Duccio Cavalieri

**Affiliations:** 1 Department of Neuroscience, Pharmacology and Child’s Health (NEUROFARBA), University of Florence, Florence, Italy; 2 Department of Experimental Medicine and Biochemical Sciences, University of Perugia, Perugia, Italy; 3 National Aspergillosis Centre and Mycology Reference Centre, University Hospital of South Manchester, University of Manchester, Manchester, United Kingdom; 4 School of Translational Medicine, Manchester Academic Health Science Centre, University of Manchester, Manchester, United Kingdom; 5 Innovation and Research Center, Edmund Mach Fondation, San Michele all’Adige (TN), Italy; University of Iowa Carver College of Medicine, United States of America

## Abstract

For over a century microbiologists and immunologist have categorized microorganisms as pathogenic or non-pathogenic species or genera. This definition, clearly relevant at the strain and species level for most bacteria, where differences in virulence between strains of a particular species are well known, has never been probed at the strain level in fungal species. Here, we tested the immune reactivity and the pathogenic potential of a collection of strains from *Aspergillus* spp, a fungus that is generally considered pathogenic in immuno-compromised hosts. Our results show a wide strain-dependent variation of the immune response elicited indicating that different isolates possess diverse virulence and infectivity. Thus, the definition of markers of inflammation or pathogenicity cannot be generalized. The profound understanding of the molecular mechanisms subtending the different immune responses will result solely from the comparative study of strains with extremely diverse properties.

## Introduction

The importance of fungal cell wall in prompting immune response has been widely investigated indicating that a multilayered cell wall controls the exposure of pathogen-associated molecular patterns to immune cells [1], [2]. So far, studies on immune responses to fungi have for the vast majority used purified antigens and eventually entire cells of reference pathogens [1], [2], [3], [4], [5], [6], [7], [8], [9], [10]. Often the use of different strains led to different conclusions on the role of fundamental mechanisms, such as those mediated by different receptors [11], [12], [13], [14], [15]. Recently, the use of *in vitro* primary cell culture systems has been proposed as a viable strategy for a first screening of mutant strains of *Candida* spp., to identify virulence traits with regard to host cell response and pathogen invasion [16].

The definition of pathogenic fungal species is also a concept whose borders have yet to be properly delineated. Fungi have developed a variety of mechanisms for evading or down-regulating the host immune response, mainly by modification of cell wall component exposure. Phenotypic switching is a strategy employed by several pathogenic fungi such as *Candida albicans*, being able to switch from a non-pathogenic cell-round shape, the yeast form, to the pathogenic hyphal form. Similarly, *Aspergillus fumigatus* encounters the immune system in a variety of developmental stages from conidia through to mature mycelium. Hyphal formation is associated with loss of proper recognition and a shift towards anti-inflammatory response or evasion mechanisms [17]. *A. fumigatus* is currently the major air-borne fungal pathogen and is generally regarded as a passive opportunistic pathogen. Production of scavenger molecules, such as melanin, mannitol, catalase and superoxido-dismutase, enables *Aspergillus spp.* to resist damage by reactive oxygen intermediates [18], [19], [20]. Thus, according to current knowledge, even the most aggressive pathogen, such as *A. fumigatus*, lacks virulence traits of the sophistication shown by those evolved in bacteria. Its ability to establish infection seems mainly due to its robustness and ability to adapt to a wide range of environmental conditions [20].

Although the anti-inflammatory potential of specific microbial strains was occasionally suggested *in vitro* [21], [22] or in animals [22], [23], few independent observations support the idea that fungal strain variability could explain differences in immune response [23], [24]. Additionally, our existing knowledge of immune response to *A. fumigatus* is derived from a wide range of isolates. To our knowledge, no studies have systematically addressed fungal immune reactivity at the strain level.

To investigate how the origin, ecological niches and phenotypic properties could affect the host immune reactivity to *A. fumigatus*, we screened a diverse set of strains for their pathogenic and immuno-modulatory performance. Selected candidate strains were then tested on animal models. This study offers new clues whether the commonly studied molecular components of these pathogens are really sufficient to account for the immune response to the live pathogen. Determining whether strain specific differences in the immune responses are either advantageous to the pathogen, or to the host, improves our understanding of host-pathogen interactions and suggests a need to rethink our understanding of the pathogenicity concept in fungi.

## Materials and Methods

### Ethics Statement

For the *in vitro* experiments using human cells, the experimental plan was approved by the local Ethical Committee of Azienda Universitaria Ospedaliera Careggi (AUOC, Careggi Hospital, Florence; Italy), and written informed consent was obtained from all donors (approval document n. 87/10). The study was designed in conformity with the international recommendation (Dir. EU 2001/20/EC) and its italian counterpart (DM 15 Luglio 1997; D.Lvo 211/2003; D.L.vo 200/2007) for clinical trial and following the Declaration of Helsinki, to assure protection and care of subjects involved. In mouse models, experiments were performed according to the Italian Approved Animal Welfare Assurance A-3143-01 and Legislative decree 157/2008-B regarding the animal licence obtained by the Italian Ministry of Health lasting for three years (2008–2011). Infections were performed under avertin anesthesia and all efforts were made to minimize suffering. As clearly described by Clemons and Stevens [25], the outcome of pulmonary aspergillosis critically depend on the inocula, such as too severe an infection result in early deaths, presumably associated with an acute inflammatory response, whereas an inoculum that is too low may result in no mortality and even clearance of the fungus. Therefore, there are no solid evidence to establish the correspondence of early clinical signs and final disease outcome in murine aspergillosis and alternative, surrogate endpoints are lacking. The experimental protocol was designed in conformity with the recommendations of the European Economic Community (86/609/CEE) for the care and the use of laboratory animals, was in agreement with the Good Laboratory Practices and was approved by the animal care Committee of the University of Perugia (Perugia, Italy).

### Fungal Strains

*A. fumigatus* strains used in the study are listed in Table 1. To obtain color mutants, spores from a single colony of Af293 were collected and subjected to UV mutagenesis. 20 ml of each spore suspension (10^7^ viable spores ml^−1^) was gently agitated by a magnetic flea in a glass petri dish (with the lid removed) 4 cm below a UV lamp (UVP, model: R-52G). A dose response experiment was carried out by removing 0.5 ml samples at 20 s intervals over a 100 s period. Irradiated spore suspensions were stored in foil-wrapped Eppendorf tubes at 4°C overnight to avoid photoreactivation. Dilutions of spore suspension were made in sterile distilled water in foil-wrapped Eppendorf tubes. Samples of spore suspension, which had been exposed to up to 60 seconds of UV irradiation, were diluted in sterile distilled water by factors of 10^3^ and 10^4^. 0.1 ml aliquots of these dilutions were spread onto Vogel’s agar plates (3 replicates per dilution) using a flame-sterilised glass spreader. Samples taken after 60 s were diluted by factors 10^1^, 10^2^ and 10^3^. All plates were incubated in the dark at 37°C. Colonies were counted initially after two days and finally after four days of incubation. A kill curve was plotted to estimate the exposure time to UV light to kill 95% of spores. This was then used for subsequent mutagenesis procedures and UV irradiated spores were kept at 4°C in a foil-wrapped universal tube. The mutagenized spores were spread onto SAB medium at a concentration to give 50–70 colonies per plate and incubated at 37°C for 2–3 days. Triton X-100 (0.1%) was included in the medium to restrict colony growth. The colour mutants were selected by visual inspection. For the melanin pathway mutants, gene knockout cassettes were constructed by modified PCR fusion method using primers shown in Table S1 [26] Primers hph_F and hph_R, which contained the PCR fusion linkers “CCGGCTCGGTAACAGAACTA” and “GGGAGCATATCGTTCAGAGC”, were used to amplify the hygromycin selectable marker, a ∼2.8 kb hygromycin B phosphotransferase cassette from pAN7-1 under the *gpdA* promoter and TtrpC terminator. The hygromycin cassette was fused to upstream and downstream flanks of *alb1*, *arp1*, *arp2*, *ayg1*, *abr2* and *rodA* to make the final knockout cassette as previously described [27]. Gene knockout cassettes were used to delete transporter genes in the CEA10 derived strain A1160 Δ*Ku80* ^−^ *pyrG^+^*. Fungal transformations were performed using previously described methodologies [27]. Transformants were selected on YPS media (2% yeast extract, 0.5% peptone, 0.9 M sucrose, 5 mM Tris, 1.5% technical agar, pH 6.0) supplemented with 200 µg/ml hygromycin B (Melford) and incubated at 37°C until the appearance of resistant colonies. Growing colonies were streaked on SAB agar supplemented with 200 µg/ml hygromycin B and a single colony from each strain was grown on the same media for DNA extraction and other subsequent analyses. Transformed colonies were screened by PCR using the flanking primers from the first step of the PCR fusion reaction (P1 and P4), which appear outside the boundary of the gene KO cassette. Primer set P1 and hph_R was used to confirm the insertion of the upstream flank whilst set hph_F and P4 was used to confirm insertion of the downstream flank. Additionally primers P9 and P10, which match the sequence of the target gene, were used to confirm gene deletion**.** *A. fumigatus* clinical isolates were obtained from the Mycology Reference Centre Manchester (MRCM). All mutants in the uridine biosynthetic pathway were isolated by selection on fluoro-orotic acid (5-FOA). *PyrG* mutation was confirmed by transforming isolates with the *AfpyrG* gene. Mutants in the nitrate assimilation pathway were isolated by selection on 600 mM potasium chlorate. *NiaD* mutants were confirmed by their ability to grow in the presence of hypoxantheine and nitrite as sole nitrogen sources but not nitrate. A complete list of the primers used for the generation of *Aspergillus* mutants is reported as Table S1.

**Table 1:** Complete list of the strains used in the study.

Name	Species	Origin	References
A4	*A. nidulans*	Manchester	Nierman et al., 2005 [44]
Af293	*A. fumigatus*	Manchester	Nierman et al., 2005 [44]
Af293 pyrG^−^	*A. fumigatus*	Manchester	this work
Af293 VKBR1	*A. fumigatus*	Manchester	this work
Af293 VKBR2	*A. fumigatus*	Manchester	this work
Af293 VKBR3	*A. fumigatus*	Manchester	this work
Af293 VKBR4	*A. fumigatus*	Manchester	this work
Af293 VKBR5	*A. fumigatus*	Manchester	this work
Af293 VKOL1	*A. fumigatus*	Manchester	this work
Af293 VKWH1	*A. fumigatus*	Manchester	this work
Af293 VKWH2	*A. fumigatus*	Manchester	this work
Af293 VKLG1	*A. fumigatus*	Manchester	this work
Af300	*A. fumigatus*	Manchester	this work
Af300 niaD^−^	*A. fumigatus*	Manchester	this work
CEA10 (CBS144-89 )	*A. fumigatus*	Paris	Beauvais et al., 1997 [45]
CEA10 pyrG^−^	*A. fumigatus*	Manchester	this work
CEA10 pyrG^−^ niaD^−^	*A. fumigatus*	Manchester	this work
alb1	*A. fumigatus*	Manchester	this work
roda	*A. fumigatus*	Manchester	this work
abr2	*A. fumigatus*	Manchester	this work
ayg1	*A. fumigatus*	Manchester	this work
arp1	*A. fumigatus*	Manchester	this work
arp2	*A. fumigatus*	Manchester	this work
F14206	*A. fumigatus*	Manchester	this work
F15767	*A. fumigatus*	Manchester	this work
F12760	*A. fumigatus*	Manchester	this work
F16216	*A. fumigatus*	Manchester	this work
F11678	*A. fumigatus*	Manchester	this work
F12041	*A. fumigatus*	Manchester	this work
F12285	*A. fumigatus*	Manchester	this work
F11628	*A. fumigatus*	Manchester	this work
F13402	*A. fumigatus*	Manchester	this work

### Human Cell Preparation and Stimulation

PBMCs were isolated from buffy coat blood sample from 6 healthy donors from the Transfusion Unit of the Careggi Hospital (Florence, Italy) by Ficoll-Hypaque density gradient centrifugation (Biochrom AG). For DC experiments, monocytes were isolated from low density PBMCs by magnetic enrichment with anti-CD14 beads (Miltenyi Biotec). Cells were cultured in the presence of GM-CSF (800 U/ml) and recombinant IL-4 (1000 U/ml) for 6 days to allow DC differentiation [28]. DC activation was induced by fungal strains in the different life stages. Depending on the experiments, moDCs were added at different concentrations. A serial dilution of live/UV-treated yeast preparations was added to the moDCs at different stimuli:DC ratios. For confirmation experiments, PBMCs from the same healthy subjects were used; stimulation was performed as in DC challenge experiments. For RT-PCR cells were collected after 24 hr or 5 days of stimulation. Supernatants were collected after 5 days of stimulation for cytokine detection.

### Fungal Infections and Treatments

For *in vitro* experiments, *A. fumigatus* hyphae or conidial preparation were performed as previously described [14]. It should be noted that in some of the experiments performed in this study, UV-killed microorganisms were used otherwise properly indicated. UV-treatment was performed on recovered cultures for 2 h. A viability assay was conducted by CFU counting after plating the cultures on agar plate post-treatment. For infection, female C57BL6 mice, 8–10 wk old, were purchased from Charles River (Calco, Italy). Mice were anesthetized by intraperitoneal (i.p.) injection of 2.5% avertin (Sigma Chemical Co, St. Louis, MO) before instillation of a suspension of 2×10^7^ viable conidia/20 µl saline intranasally (i.n.). Fungi were suspended in endotoxin–free (Detoxi–gel; Pierce, Rockford, IL) solutions (<1.0 EU/mL, as determined by the LAL method). Mice were monitored for fungal growth in lung and brain (CFU/organ, mean ± SE), survival (MST, days), histopathology (Periodic acid–Schiff (PAS)-staining of sections of paraffin–embedded tissues) and patterns of cytokine/chemokine gene expression and production. No other clinical signs could be recorded, despite the fact that the brain infection with *Aspergillus* is the most common extra-pulmonary site of infection, and one that results in about 80% mortality [25]. Bronchoalveolar lavage (BAL) was performed by cannulating the trachea and washing the airways with 3 ml of PBS to collect the BAL fluid. Total and differential cell counts were done by staining BAL smears with May–Grünwald Giemsa reagents (Sigma) before analysis. At least 200 cells per cytospin preparation were counted and the absolute number of each cell type was calculated. Photographs were taken using a high–resolution Microscopy Olympus DP71 (Olympus, Milan, Italy).

### Cytokine Production

At the indicated times, supernatants from human cell cultures were collected and cytokine detection was performed using the Milliplex® MAP human cytokine/chemokine kit (Millipore), according to the manufacturer’s instructions. Cytokine levels on lung homogenates from infected mice were determined a week after the infection by enzyme-linked immunosorbent assays (BD Biosciences Pharmingen, R&D Systems and eBioscience). The detection limits (pg ml) of the assays were <10 for IFN-γ, <3 for IL-10, <10 for IL-17A.

### Real–time PCR

Human total RNA was extracted with the Rneasy Mini Kit (Qiagen, Milan, Italy) from human PBMCs derived from 6 distinct donors. Random hexamers and reverse transcriptase kit (SuperScript II, Invitrogen) were used for cDNA synthesis. Transcripts for *IL-6*, *IL-10, TNA, IL1B, IL-17A,* and *IFNG* genes were quantified with Applied Biosystems predesigned TaqMan Gene Expression Assays and reagents according to the manufacturer’s instructions. Quantification of the PCR signals was performed by comparing the cycle threshold (Ct) value of the gene of interest with the Ct value of the reference gene *GAPDH*. Values are expressed as fold increase of mRNA relative to that in unstimulated cells. Murine total RNA was extracted from lungs using RNeasy Mini Kit and was reverse transcribed with Sensiscript Reverse Transcriptase (Qiagen) according to the manufacturer’s directions. The sense/antisense primers were as described [29]. Real–time RT–PCR was performed using the iCycler iQ detection system (Bio–Rad) and SYBR Green chemistry (Finnzymes Oy, Espoo, Finland). Quantification of the PCR signals was performed by comparing the cycle threshold (Ct) value of the gene of interest with the Ct value of the reference gene GAPDH. Values are expressed as fold increase of mRNA relative to that in unstimulated cells.

### Statistics

For the DC experiments, statistically significance was evaluated using a Mann-Whitney test and a post-hoc comparison. Results, expressed as p-value, are reported for each cytokine measurement and for each strain comparison in Table S2. For human PBMCs and mice data, a paired t-test comparison was used.

## Results

### Immune-based Diversity of Different Common Laboratory Aspergillus Strains

Several strains of *A. fumigatus* originally isolated from a clinical setting are in regular use as laboratory strains, these include the sequenced strain Af293, CEA10 and Af300 (Table 1).

**Figure 1:**
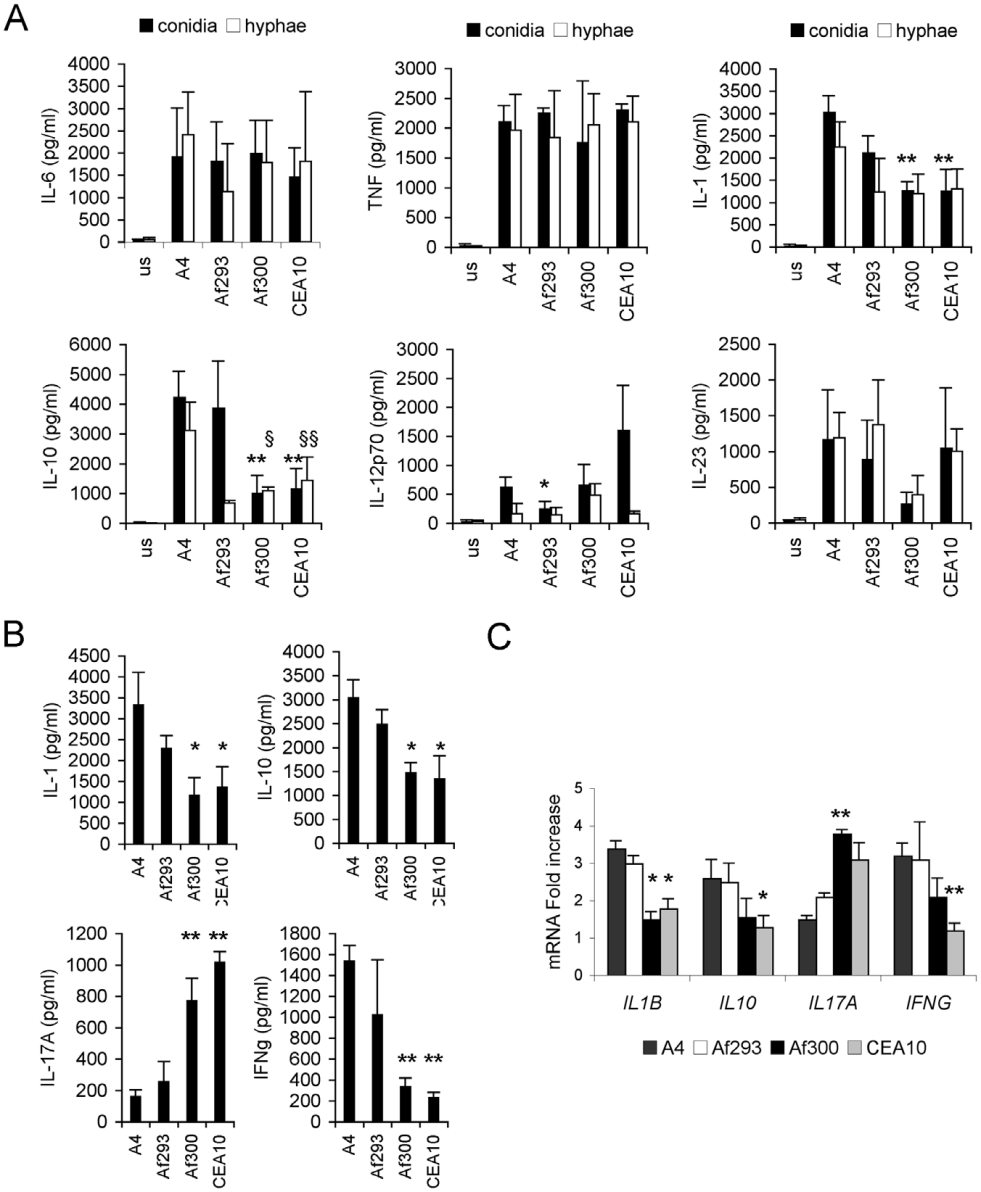
Immune response to different WT *Aspergillus* strains. Different WT strains elicit diverse inflammatory responses and prime peculiar adaptive Th response. (A) DCs were cultured with UV-killed conidia or hyphae of WT strains for 24 hours or without any stimuli (unstimulated, us) and supernatants were used for TNFα, IL-10, IL-1β, IL-6, IL-12p70 and IL-23 measurements. Data are represented as mean+SD (N = 6), *p≤0.05, **p≤0.01, INF^−^ conidia *vs* INF^+^conidia; ^§^p≤0.05, ^§§^p≤0.01, INF^−^ hyphae *vs* INF^+^ hyphae. Complete statistically significant p-values are collected in Table S2. (B,C) Healthy PBMCs were cultured with live conidia or without any stimulus, and cytokine protein (A) and transcript (B) levels were measured. Data are represented as mean+SD (N = 6), *p≤0.05, **p≤0.01, INF^−^ *vs* INF^+^ strains.

The cytokine induction pattern for these 3 laboratory strains along with an *A. nidulans* ‘laboratory’ isolate was evaluated by an *in vitro* assay challenging monocyte derived DCs from 6 distinct healthy donors with either hyphae or fungal conidia. Pattern recognition receptors expressed on these immune cells contribute to the specific detection of fungi [30], [31]. Direct contact with fungi leads to the maturation of DCs, which is characterized by an increase in antigen presentation, expression of costimulatory molecules and cytokine production. In our assays, tumor necrosis factor alpha (TNF-α) and interleukin (IL)-10, IL-1ß, IL-6, IL-12p70 and IL-23 levels were measured (Figure 1A). This investigation indicate that a large diversity in the immuno-modulatory profiles exists among *A. fumigatus* strains as quantified directly by cytokine comparison, rather than the traditional IL-10/IL-12 ratio [32], [33], which obviously is inappropriate here to describe the high complexity of the fungal morphotype-dependent immune response [33]. Statistical analysis indicated that the strain-dependent cytokine variation was significantly different among the strains tested (Figure 1A and Table S2). Release of pro-inflammatory mediators, such as TNF-α was very high; by contrast, a weak induction of IL-12p70 was observable irrespective of the developmental stage of the fungus (Figure 1A). In addition to the high donor variability observed, the screening revealed a highly strain-dependent induction of IL-10 and IL-1 ß. *A. nidulans* A4 and *A. fumigatus* Af293 strain elicited a high induction of IL-10 and IL-1ß while *A. fumigatus* Af300 and CEA10 were less potent inducers of these cytokines. We extended our examination of the strains to include the response of PBMCs to live fungal cells. Consistently with the previous results Af300 and CEA10 elicited a higher inflammatory response whereas Af293 and A4 did not (Figure 1B–C). To correlate the cytokine profiles with the ability of these strains to prime for T_H_1 or T_H_17 adaptive immune responses, human PBMCs were stimulated with live strains and both gene transcripts for *IFNG* and *IL17A* and their cytokine levels were evaluated on stimulated PBMCs (Figure 1B–C). The WT Af293 and A4 strains induced more INF-γ and less IL-17A hence suggesting initiation of a T_H_1 response; by contrast the WT Af300 and CEA10 strains induced more IL-17A. We will indicate them as “inflammatory” (Af293 and A4) and “hyper-inflammatory” (Af300 and CEA10) along the text.

### The Different WT Strains Induce Disparate Inflammatory Responses in Mice

**Figure 2:**
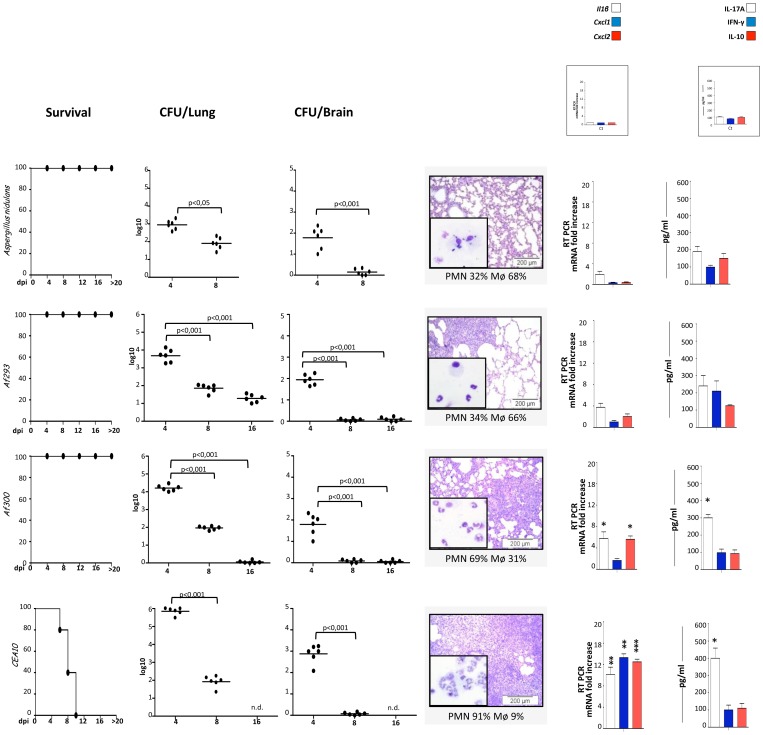
*In vivo* immune reactivity to *A. fumigatus* WT strains. C57BL/6 mice were infected intranasally with different strains of *A. fumigatus* (6 mice/group). Survival (%), fungal growth (mean log_10_ CFU ± SE, N = 3) in the lungs and brains of infected mice were assessed at different days post-infection (dpi). The CFUs between wild-type and the corresponding mutant strains were statistically significant (p values ranging from ≤0.01 to ≤0.001). Lung histology (PAS staining) and BAL morphometry [%, mean ± SD, of mononuclear (MNC) or polymorphonuclear (PMN) cells] were done at 4 dpi. Representative images of two independent experiments were depicted; bars indicate magnifications. Total lung RNA was extracted at 4 dpi and the relative expression of *Il1β*, *Cxcl1* and *Cxcl2* genes was assessed by RT-PCR. Lung homogenates at 4 dpi were tested for levels of IL-17A, IFN-γ, and IL-10 by specific ELISA (mean values ± SD, N = 3). *p≤0.05, **p≤0.01 and ***p≤0.001, wild-type strains *vs* uninfected mice.

The majority of virulence studies that assessed the pathogenicity of *Aspergillus* strains have been performed in immunosuppressed models. We assessed the pathogenic potential of the different fungal strains *in vivo* in immunocompetent C57BL/6 mice, whose immunological response to the fungus is well characterized [34], [35]. Mice were intranasally inoculated with the different fungal strains and parameters of infection were evaluated in terms of survival, fungal growth in the lung and dissemination into the brain, histopathology and cytokine/chemokine gene expression and production. As reported in Figure 2, important differences were observed among the different WT strains (two “hyper-inflammatory”, i.e, AF300 and CEA10 and two ^“^inflammatory”, i.e., *A. nidulans* and Af293). All mice, except those infected with CEA10, survived the infection. The fungal growth in the lung and brain was apparently higher, early in infection, in mice infected with the “hyper-inflammatory” as compared to the inflammatory strains; however, clearance of the fungus was higher in mice infected with the “hyper-inflammatory” as compared to the other^−^ strains. The higher fungal burden and increased fungal clearance was associated with a noticeable inflammatory pathology in the lung of mice infected with the “hyper-inflammatory” strains, as revealed by increased infiltration of inflammatory cells, particularly neutrophils, and the increased expression of *Cxcl1* and *Cxcl2* chemokines. Of interest, the levels of inflammatory/anti-inflammatory cytokines strictly mirrored those observed *in vitro*, as the production of IL-1ß and IL-17A were locally higher in mice infected with the “hyper-inflammatory” strains. Neither IFN-γ nor IL-10 was instead elevated in mice infected with the Af300 and CEA10 strains. These data indicate that different inflammatory responses, likely affecting the outcome of the infection, are elicited by different *Aspergillus* WT strains *in vivo.*

### Effects of Mutation Introduction on the Immune Response Elicited by Different WT Strains

**Figure 3:**
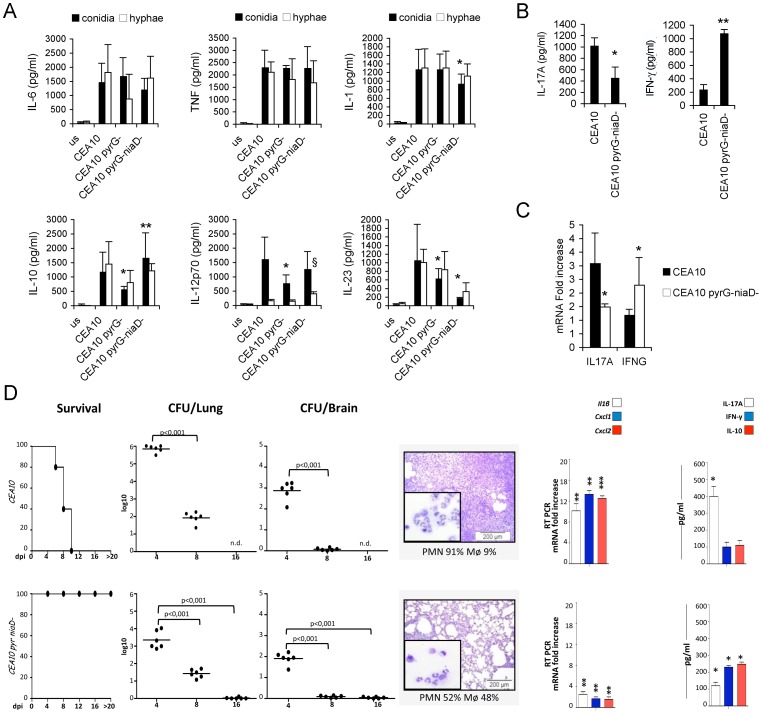
Immune response to different *Aspergillus* mutant strains. Different mutant strains elicit diverse inflammatory responses and prime peculiar adaptive T_H_ response. (A) DCs were cultured with UV-killed conidia or hyphae for 24 hours or without any stimuli (unstimulated, us) and supernatants were used for TNFα, IL-10, IL-1β, IL-6, IL-12p70 and IL-23 measurements. Data are represented as mean+SD (N = 6), *p≤0.05, **p≤0.01, conidial mutant strains *vs* the conidial WT strain; ^§^p≤0.05, ^§§^p≤0.01, hyphal mutant strains vs hyphal WT strain. Complete statistically significant p-values are collected in Table S2. (B,C) Healthy PBMCs were cultured with live conidia or without any stimulus, and cytokine protein and transcript levels were measured. Data are represented as mean+SD (N = 6), *p≤0.05, **p≤0.01, mutants strain *vs* WT strain. (D) C57BL/6 mice were infected intranasally with different strains of *A. fumigatus* (6 mice/group). Survival (%), fungal growth (mean log_10_ CFU ± SE, N = 3) in the lungs and brains of infected mice were assessed at different days post-infection (dpi). The CFUs between wild-type and the corresponding mutant strains were statistically significant (p-values ranging from ≤0.01 to ≤0.001). Lung histology (PAS staining) and BAL morphometry [%, mean ± SD, of mononuclear (MNC) or polymorphonuclear (PMN) cells] were done at 4 dpi. Representative images of two independent experiments were depicted; bars indicate magnifications. Total lung RNA was extracted at 4 dpi and the relative expression of *Il1β*, *Cxcl1* and *Cxcl2* genes was assessed by RT-PCR. Lung homogenates at 4 dpi were tested for levels of IL-17A, IFN-γ, and IL-10 by specific ELISA (mean values ± SD, N = 3). *p≤0.05, **p≤0.01 and ***p≤0.001, wild-type strains *vs* uninfected mice or mutants strains *vs* the wild-type strain.

To examine the effects of attenuation of virulence on the murine response we used isolates deficient in UMP biosynthetic pathway in the CEA10 genetic background. Introducing *pyrG* ^−^ and *pyrG niaD* ^−^ mutations causes auxotrophy for uridine and uracil and as a result, mutant strains are unable to establish an infection in a murine host [36]. We found that in our genetic backgrounds, UV-killed spores of both mutants induce a similar-or even stronger-inflammatory response *in vitro* compared to that prompted by the respective WT counterpart (Figure 3A). When live cells were used to challenge PBMCs, CEA10 *pyrG* ^−^ *niaD* ^−^, which is unable to germinate, induced high levels of production of IL-10 and INF-γ and low levels of IL-17A modulating the T_H_17 response of the wild-type isolate to a T_H_1 response. (Figure 3B–C). Interestingly, the levels of inflammatory cytokines were higher after DC stimulation with the Af300 *niaD* strain as compared to the WT strain, as also indicated by the high level of IL-17A (Figure 3B–C).

**Figure 4:**
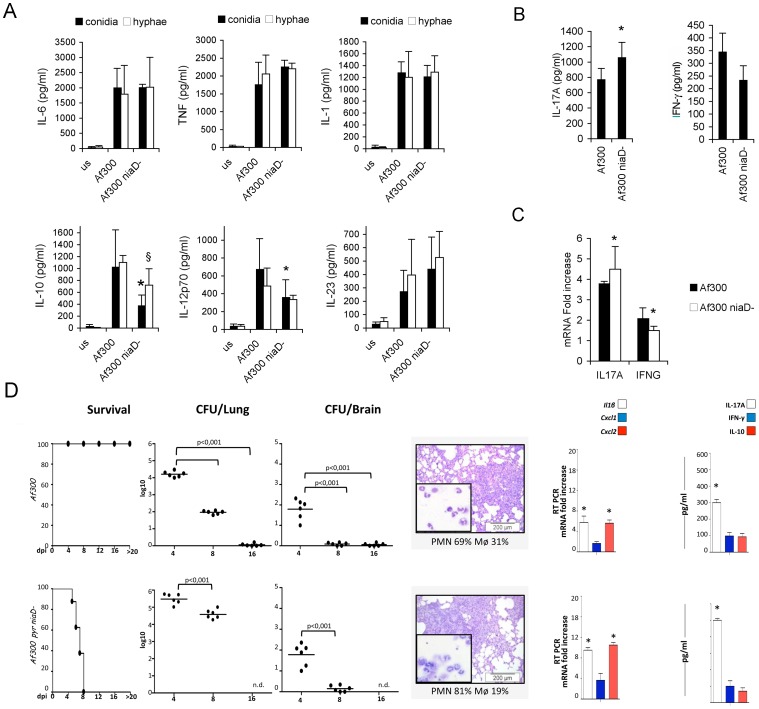
*In vivo* immune response to *A. fumigatus* mutant strains. Different mutant strains elicit diverse inflammatory responses and prime peculiar adaptive T_H_ response. (A) DCs were cultured with UV-killed conidia or hyphae for 24 hours or without any stimuli (unstimulated, us) and supernatants were used for TNFα, IL-10, IL-1β, IL-6, IL-12p70 and IL-23 measurements. Data are represented as mean+SD (N = 6), *p≤0.05, **p≤0.01, conidial mutant strains *vs* the conidial WT strain; ^§^p≤0.05, ^§§^p≤0.01, hyphal mutant strains vs hyphal WT strain. Complete statistically significant p-values are collected in Table S2. (B,C) Healthy PBMCs were cultured with live conidia or without any stimulus, and cytokine protein and transcript levels were measured. Data are represented as mean+SD (N = 6), *p≤0.05, **p≤0.01, mutants strain *vs* WT strain. (D) C57BL/6 mice were infected intranasally with different strains of *A. fumigatus* (6 mice/group). Survival (%), fungal growth (mean log_10_ CFU ± SE, N = 3) in the lungs and brains of infected mice were assessed at different days post-infection (dpi). The CFUs between wild-type and the corresponding mutant strains were statistically significant (p values ranging from ≤0.01 to ≤0.001). Lung histology (PAS staining) and BAL morphometry [%, mean ± SD, of mononuclear (MNC) or polymorphonuclear (PMN) cells] were done at 4 dpi. Representative images of two independent experiments were depicted; bars indicate magnifications. Total lung RNA was extracted at 4 dpi and the relative expression of *Il1β*, *Cxcl1* and *Cxcl2* genes was assessed by RT-PCR. Lung homogenates at 4 dpi were tested for levels of IL-17A, IFN-γ, and IL-10 by specific ELISA (mean values ± SD, N = 3). *p≤0.05, **≤0.01 and ***p≤0.001, wild-type strains *vs* uninfected mice or mutants strains *vs* the wild-type strain.

*In vivo* experiments confirmed the *in vitro* results. Assessment of virulence of the CEA10 *pyrG niaD* ^−^ strain in the murine model confirmed it was unable to cause mortality (Figure 3D). The fungal growth and the host inflammatory response were indeed reduced, as revealed by histopathology of the lung and expression of inflammatory cytokines and chemokines as compared to mice infected with the virulent CEA10 WT strain (Figure 3D). Interestingly, the virulence of a *niaD* ^−^ mutant in a different genetic background, such as Af300 strain, was instead increased, as revealed by the *in vitro* inflammatory response (Figure 4A–C) as well as the decreased survival, unrestrained fungal growth and high levels of inflammatory cytokine production *in vivo* (Figure 4D). However, in this context, it is unlikley that the nitrate reductase gene (*niaD*) is responsible for this change in phenotype. The mutation of the *niaD* gene in this strain is as a result of a chromosomal translocation and as such other mutations are present in this strain. It is unlikley that loss of nitrate reductase would lead to an increase in virulence, therefore another as yet undefined mutation in the strain is probably contributing to this phenotype. Nevertheless, such result indicates once more how mutations/translocations normally occurring in nature could profoundly affect virulence and immuno-modulatory properties.

### Melanization, Cytokine Release and Virulence

**Figure 5:**
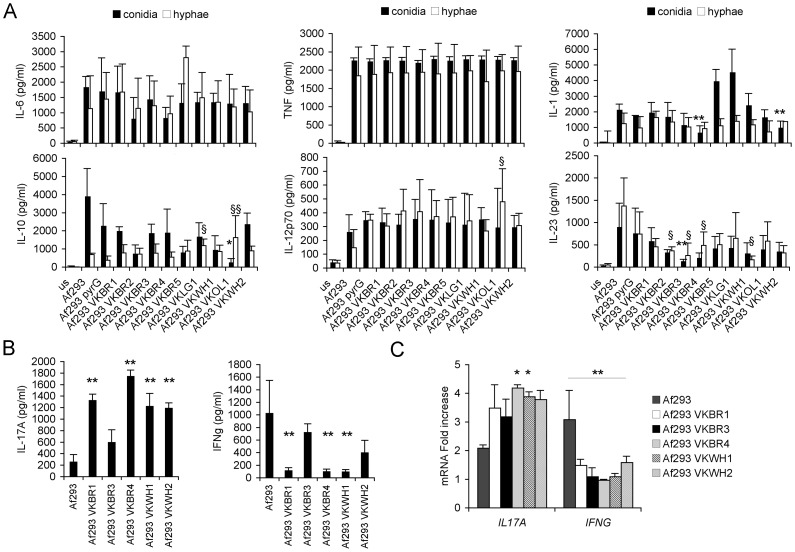
Immune response to different *Aspergillus* color mutants. Different mutant strains elicit diverse inflammatory responses and prime peculiar adaptive T_H_ response. (A) DCs were cultured with UV-killed conidia or hyphae for 24 hours or without any stimuli (unstimulated, us) and supernatants were used for TNFα, IL-10, IL-1β, IL-6, IL-12p70 and IL-23 measurements. Data are represented as mean+SD (N = 6), *p≤0.05, **p≤0.01, conidial mutant strains *vs* conidial WT strain; ^§^p≤0.05, ^§§^p≤0.01, hyphal mutant strains vs hyphal WT strain. Complete statistically significant p-values are collected in Table S2. (B,C) Healthy PBMCs were cultured with live conidia or without any stimulus, and cytokine protein and transcript levels were measured. Data are represented as mean+SD (N = 6), *p≤0.05, **p≤0.01, mutants strain *vs* WT strain.

**Figure 6:**
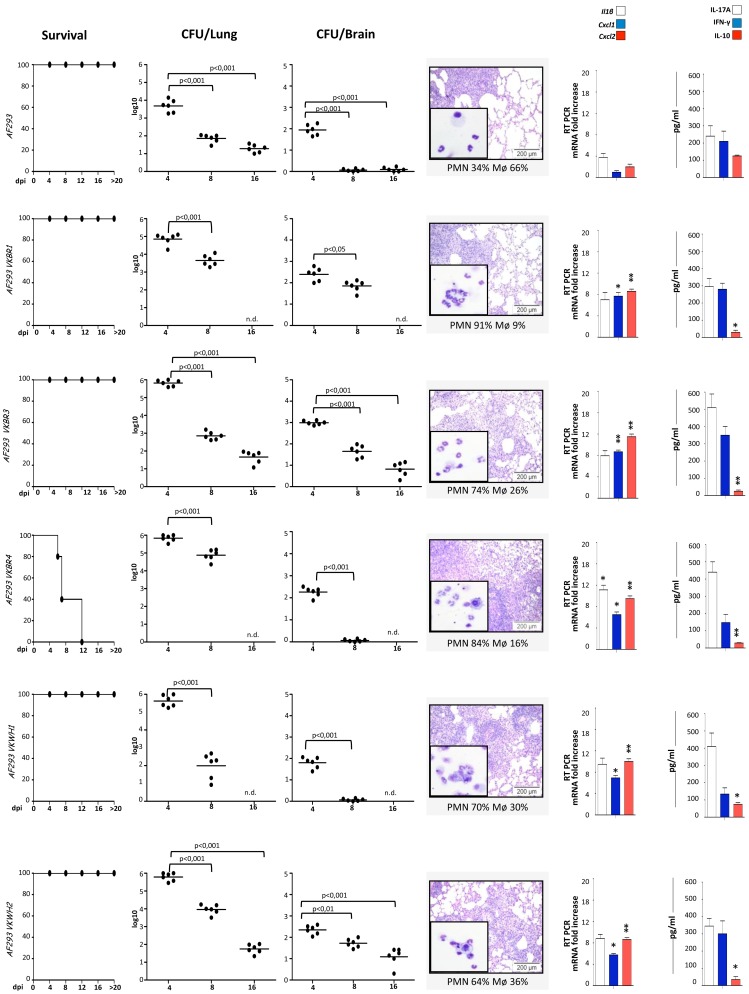
*In vivo* immune response to *A. fumigatus* color mutants. C57BL/6 mice were infected intranasally with different strains of *A. fumigatus* (6 mice/group). Survival (%), fungal growth (mean log_10_ CFU ± SE, N = 3) in the lungs and brains of infected mice were assessed at different days post-infection (dpi). The CFUs between wild-type and the corresponding mutant strains were statistically significant (p values ranging from ≤0.01 to ≤0.001). Lung histology (PAS staining) and BAL morphometry [%, mean ± SD, of mononuclear (MNC) or polymorphonuclear (PMN) cells] were done at 4 dpi. Representative images of two independent experiments were depicted; bars indicate magnifications. Total lung RNA was extracted at 4 dpi and the relative expression of *Il1β*, *Cxcl1* and *Cxcl2* genes was assessed by RT-PCR. Lung homogenates at 4 dpi were tested for levels of IL-17A, IFN-γ, and IL-10 by specific ELISA (mean values ± SD, N = 3). *p≤0.05, **p≤0.01 and ***p≤0.001, wild-type strains *vs* uninfected mice or mutants strains *vs* the corresponding wild-type strain.

Melanization in *A. fumigatus* confers bluish-grey color to conidia. Previous studies have observed an association with the melanization of the conidial cell wall, the ability to elicit an immunological response from human PBMCs, and increase in strain pathogenicity [18]. To further investigate how the phenotypic properties could affect the host immune reactivity to *A. fumigatus*, we screened a set of color mutants for their immuno-modulatory performance. In particular, 9 *A. fumigatus* Af293 color mutant strains were obtained by UV mutagenesis as described in Material and Methods and selected for their different colony colors (WH, white; BR, brown). After DC stimulation, all the mutants induce a similar -or even stronger- inflammatory response to that prompted by their respective WT counterpart as indicated by the different pattern released by either the conidial or the hyphal form of the strains (Figure 5A). Af293 conidia elicit a strong IL-10 production, while its color mutant strains revealed a trend to lower induction of IL-10. In particular, while some strains induce high level of IL-10 (Af293 VKWH2, VKBR1, VKBR3 and VKBR4) others do not (VKBR2, VKBR5, VKWH1 and VKOL1). A significant reduction in IFN-γ production and a concordant increase of IL-17A level was observed when PBMCs were challenged with live conidia from the 5 strains exhibiting a pro-inflammatory response from DCs (Figure 5B and 5C). The murine infection model further confirmed the inflammatory changes induced by the spore colour mutants as compared to the WT strain, as indicated by the elevated fungal burden and fungal persistence, dissemination into the brain and pattern of tissue inflammation and inflammatory response (Figure 6). Quite unexpectedly, among the strains inducing the worsen outcome, we identified the Af293 VKWH1 and Af293 VKWH2 strains, characterized, respectively, by a sandy and an albino conidial phenotypes.

**Figure 7:**
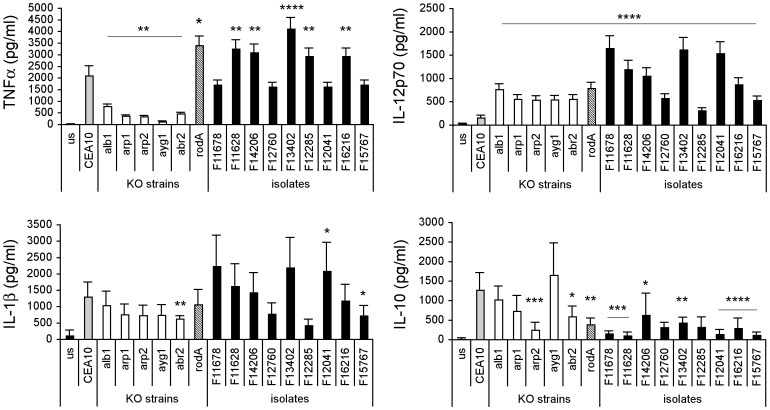
Effect of melanin content in the immune reactivity of *A. fumigatus* clinical isolates. Ability of DC to discriminate among different melanin color mutants and clinical isolates of the *Aspergillus* species was tested as differential cytokine production. DCs were cultured with live conidia of wt CEA10 strain, different knock out (KO) mutants, Aspergillosis isolates or without any stimulus (unstimulated, us) for 24 hours and supernatants used for TNFα, IL-10, IL-1β, IL-6, IL-12p70 and IL-23 measurements. *p≤0.05, **p≤0.01 and ***p≤0.001, ****p≤0.0001, mutants strains *vs* CEA10 strain, isolates *vs* CEA10 strain.

The increase in inflammation caused by spore colour mutants derived from a strain deemed to be anti-inflammatory led us to investigate the role of pigmentation in a strain already thought to be pro-inflammatory (CEA10). We generated gene knockouts in the *A. fumigatus* melanin biosynthetic cluster genes (*alb1*, *arp2*, *arp1*, *abr2* and *ayg1*) [29], [30], [31], [32], [33] and used live conidia to challenge human DCs. In parallel we assessed the DC cytokine responses to a strain deficient in the rodlet/hydrophobin layer (*rodA* knock out mutant) [37]. In general, we observed a reduction in the inflammatory cytokine response from the melanin mutants. All of the knockouts showed a significant reduction in their capacity to induce TNF-α whilst displaying an increase in their ability to induce IL-12p70. RodA normally encapsulates dormant spores, its removal results in the exposure of underlying proteins on the spore surface. It has been demonstrated that dormant conidia of a *rodA* mutant induces high levels of the pro-inflammatory cytokine TNF-α and IL-12p70 from human DCs when compared to the wild-type isolate (Aimanianda et al 2009). Our results show this is apparent even when a live *rodA*Δ mutant is used as a challenge to DCs suggesting that the rodlet layer has a role in shielding proteins in hyphae as well as conidia (Figure 7). In contrast to Aimanianda *et al*, but in keeping with a pro-inflammatory response, we observed a reduction in the production of IL-10. Since in nature, a great variety of colored strains exists, we compared the cytokine response to the mutant strains to the one induced by 9 *A. fumigatus* isolates from patients with chronic aspergillosis in an attempt to see if any variability exists in the immune reactivity of the isolates (Figure 7). The conidia from these strains exhibited a range of spore colour phenotypes, varying from brown conidia (F15767, F16215) to brownish-green conidia (F12041, F12285), from sand colonies (F13402, F14206) to colonies sand-coloured at the edge and green in the centre (F12760), to almost white conidia (F11628 and F11698), possibly indicating a different melanin content. We saw an exceptionally wide variability in the cytokine response to the clinical isolates. Despite several isolates show similarity with the knockout mutants, we could not perfectly match the pro-inflammatory phenotype of the isolates with the colour feature. As an example, *rodA* strain and F15767 present similar color phenotype (data not shown) and are both inflammatory as indicated by the low IL-10 induction. By contrast, even if F11628 strain presents white conidia as *alb1* strain, it induces lower level of IL-10 with respect to the albino strain. Thus, there was no discernible pattern to the way in which the DCs responded to the different strains except that all strains induced low levels of the anti-inflammatory IL-10 and a higher production of pro-inflammatory cytokines such as TNF-α, IL-1β and IL-12p70 (Figure 7). The overall results indicate that pigmentation is not a clear indicator of the strain pathogenicity as it may contribute very little in some strains but more in others.

## Discussion

In the bacterial kingdom it is well recognised that certain members of a species can vary in their ability to cause disease. However fungi are less well studied and it is assumed that all members of a species have equal ability to cause disease or induce immune responses. As a consequence, there has been little effort to standardise strains used for these studies in different laboratories.

Emerging fungal pathogens are unexpectedly becoming an ever-increasing threat in the world, in particular in immune compromised patients [38], [39], [40]. Because of the poor outcome of current treatments of invasive fungal infection, it is imperative to understand the nature of fungal pathogenesis and to develop more effective therapies for combating invasive fungal infection in humans. Given the importance to studying the nature of fungal pathogenesis, it becomes necessary to explore immune-based diversity of strains derived from different ecological niches. Several studies worldwide have attempted to address pathogenicity mechanisms using different strains as a model. It is known that different isolates of *A. fumigatus* possess divergent genomes and gene complements [41].

In this study we aimed at probing the DC immune response to the opportunistic fungal species *Aspergillus*, allowing an *in vitro* preliminary classification and characterization of different strains according to their immune reactivity. To this end, we categorized the strains based on the cytokine secretion profile of *Aspergillus*-stimulated DCs. Confirmation experiments were performed using PBMCs and the ability of particular strains to induce inflammation were further tested in mice.

In addition to the high donor variability observed, the screening revealed a strong strain-specific variation of the *in vitro* cytokine induction profiles after stimulation of immuno-competent cells, highlighted by the variable levels of IL-10 and IL-1β. For all the strains tested, independently to their cell form (conidia or hyphae), release of pro-inflammatory mediators, such as TNF-α, was very high; by contrast a weak induction of IL-12p70 was observable either upon conidial or hyphal stimulation. Statistical analysis indicated that the strain-dependent cytokine variation was significantly different among the strains tested (Table S2) and differences between blood donors did not affect the comparison of the strains based on their cytokine profiles as the patterns of DC responses to various strains were consistent from one donor to another. This investigation confirmed the large diversity in the immuno-modulatory profiles among *A. fumigatus* strains.

Despite the fact that the *in vitro* assay does not clarify the physiological mechanism(s) involved, it is well accepted as a proxy of how the immune system may sense the fungal strains and consequently polarise the immune response. Strains leading to a high IL-10/IL-12 ratio would more easily slow down an early T_H_1 response, while a high IL-10/IL-23 ratio would counterbalance an early T_H_17 response. In accordance to this hypothesis we found differences in the *in vivo* inflammatory potential of the tested strains. These differences were markedly observable upon DC stimulation with WT strains, allowing us to categorize the strains as ”inflammatory” or “hyper-inflammatory”. The observation that diverse WT strains elicit opposite responses suggest that the use of various WT strains could therefore compromise a correct understanding of the recognition mechanisms, usually based on the introduction of gene mutations. As such, the mutants derived from the “hyper-inflammatory” CEA10 and Af300 WT strains induce different immune responses either *in vitro* or *in vivo*. This observation highlights how genetic adaptation of the fungus to the host environment could influence its virulence and infectivity. Understanding the determinants contributing to the different responses of WT strains is the major follow up to this study, yet requires a substantial investment in resources and is currently under active investigation. The observation that the presence of a mutation could lead to a complete opposite outcome stresses even more the need to consider the strain specific variation traits.

Among the mutant strains tested, some of them resulted highly virulent *in vivo*. Despite Af293 mutants were all UV-induced colour mutants in the same genetic background, the albino Af293 VKWH1 strain and the brownish VKBR4 strain induced strong morbidity and, in the case of Af293 VKBR4, high mortality *in vivo*.

As mentioned before, the surface layer on the dormant *Aspergillus* conidia masks their recognition by the immune system and hence prevents immune response. In particular, the hydrophobic rodlet and melanin layers immunologically silence airborne moulds [37]. When we compared KO strains in the melanin pathway and clinical *Aspergillus* isolates with supposedly analogous spore colour morphologies, we observed a great variability in the response. Our results suggest that, despite conferring obvious virulence, melanin content could not solely account for the fungal pathogenicity, since some *Aspergillus* isolates or the sandy-albino Af293 VKWH2 and VKWH1 strains, induce a strong inflammatory response either *in vitro* or *in vivo* whereas other colour mutants do not. It is likely that rather than species-specific traits, several strain-specific traits triggered and evolved under different environmental conditions. This could, ultimately, confer different potential pathogenic or immunostimulatory traits as suggested by the different responses observed even between isolates from the same clinical condition such as Af300, Af293 and CEA10.

We would like to suggest that pathogenic traits evolved as result of rapid convergent evolution and adaptation to different ecological niches present in the host. Recent work showed that changes in carbon source altered the resistance of *C. albicans* to antifungal drugs and osmotic and cell wall stresses, thus influencing the virulence of *C. albicans* [42], [43]. Fungal strains could have evolved within the host the ability to escape immune recognition. This theory could explain the gradient in the immune responses observed as continuum in which passenger environmental fungi enter the human body, become colonizers establishing a commensal interaction and, upon failure of immune surveillance, can become invaders, and potentially seriously harm the host. We are actually testing this hyhotesis by enlarging the set of strains and comparing them using genome wide association studies. The greater resistance of *A. fumigatus* to environmental stress and its ability to be inhaled may explain his prominent role as a pathogen upon immune suppression.

We show the importance of addressing the immune reactivity of different strains and evaluating differences among strains of the same species in addition to the inter-individual variation. We also show the importance in future studies to refer to pathogenic and commensal strains rather than pathogenic and commensal species. The wide strain-dependent variation of immunological signatures suggest that the definition of markers of inflammation or pathogenicity should be reassessed and based on the understanding of strain specific armouries determining the success of host invasion. The comparative genomics and genetic study of strains carefully selected for their pro- or anti-inflammatory properties will assist further investigation of the mechanism(s) by which specific strains signal to the host and of the development of specific targeted therapy.

## Supporting Information

Table S1 **List of the primers used for the generation of** ***Aspergillus*** **mutants.** The listed primers have been used to generate the different mutants strains used in the study.
(DOC)

Table S2 **Complete statistics on the data related to the human DCs challenge experiments.** Mann-Whitney results expressed as p-value are reported for each cytokine measurement and a post-hoc comparison was performed for each couple of strains. Statically significant p-values are highlighted in colors.
(XLS)
