# Supplementary material for: Strain Dependent Variation of Immune Responses to A. fumigatus: Definition of Pathogenic Species
Source: PLoS One. 2013 Feb 18;8(2):e56651. doi: 10.1371/journal.pone.0056651 (PMC3575482; doi:10.1371/journal.pone.0056651)
Supplement: Table S1 — List of the primers used for the generation of Aspergillus mutants. The listed primers have been used to generate the different mutants strains used in the study. (DOC) [file pone.0056651.s001.doc]

| **Primer** | **Target gene** | **Primer sequence** |
| --- | --- | --- |
| 2g17530_P1 | *Abr2* | GGA CAC CGT AGT TGG TCG AT (20 bp) |
| 2g17530_P2 | *Abr2* | TAG TTC TGT TAC CGA GCC GGA AGA GAC AAC CGG CAC AAT C (40 bp) |
| 2g17530_P3 | *Abr2* | GCT CTG AAC GAT ATG CTC CCC GCA TAC GTC ATT GAT GGA G (40 bp) |
| 2g17530_P4 | *Abr2* | GTG TCA AAG CCC TCG TTG AT (20 bp) |
| 2g17530_P5 | *Abr2* | TAT CTG CAG CGC GTT TTA TG (20 bp) |
| 2g17530_P6 | *Abr2* | AAG AAC CGA CAT TTG CTC GT (20 bp) |
| 2g17540_P1 | *Abr1* | GAC TAC GGG AGC CGT AAC AC (20 bp) |
| 2g17540_P2 | *Abr1* | TAG TTC TGT TAC CGA GCC GGG TCG GTC GTG TGA GAG ACT G (40 bp) |
| 2g17540_P3 | *Abr1* | GCT CTG AAC GAT ATG CTC CCA AAG GCG AGA GGA AGT GTC A (40 bp) |
| 2g17540_P4 | *Abr1* | GGA TAG CGT GCT GAG CTA CC (20 bp) |
| 2g17540_P5 | *Abr1* | TCC ACT GGA CAC AAA CCA TC (20 bp) |
| 2g17540_P6 | *Abr1* | TCC GGG ATG CCA CTA TTA TC (20 bp) |
| 2g17550_P1 | *Ayg1* | GAC ATT GGC AAG GTG GTC TC (20 bp) |
| 2g17550_P2 | *Ayg1* | TAG TTC TGT TAC CGA GCC GGC GTG CAG TGG TTT AGC TTC A (40 bp) |
| 2g17550_P3 | *Ayg1* | GCT CTG AAC GAT ATG CTC CCT GCG CAT AGC ATT AGC CAT A (40 bp) |
| 2g17550_P4 | *Ayg1* | GCG CCA ATA ACC CAG ATA AG (20 bp) |
| 2g17550_P5 | *Ayg1* | GCC AGA TCA TCC CTG TCA AT (20 bp) |
| 2g17550_P6 | *Ayg1* | CAT ATC GAA TGG CAC GTC AC (20 bp) |
| 2g17560_P1 | *Arp2* | TGG GTC GTT ATG AGG GTG TT (20 bp) |
| 2g17560_P2 | *Arp2* | TAG TTC TGT TAC CGA GCC GGT TCA GGG TGT TTG CTT AGG G (40 bp) |
| 2g17560_P3 | *Arp2* | GCT CTG AAC GAT ATG CTC CCC GGA GTG TAT GGT TGC ATT G (40 bp) |
| 2g17560_P4 | *Arp2* | CGG ATT TTG AAC ACT GAC GA (20 bp) |
| 2g17560_P5 | *Arp2* | CGG AAG ACA TCC TCG AAT TG (20 bp) |
| 2g17560_P6 | *Arp2* | CGT GCA GTG GTT TAG CTT CA (20 bp) |
| 2g17580_P1 | *Arp1* | GGC AAT TGA GGA CAT GAG GA |
| 2g17580_P2 | *Arp1* | TAG TTC TGT TAC CGA GCC GGT TTT CGA CCA TTG TGG TGT G |
| 2g17580_P3 | *Arp1* | GCT CTG AAC GAT ATG CTC CCT TTC ATG TGA GAC AGC AAG GA |
| 2g17580_P4 | *Arp1* | CTA CTG CAA ATC GGG AAA GG |
| 2g17600_P1 | *Alb1* | TGC CAC CAA TGA GCA CTA CT |
| 2g17600_P2 | *Alb1* | TAG TTC TGT TAC CGA GCC GGG CTC TGG GAG ATC AGA TTG C |
| 2g17600_P3 | *Alb1* | GCT CTG AAC GAT ATG CTC CCC TAG GTT TGG GGG TGG AGT T |
| 2g17600_P4 | *Alb1* | AGC CCT CGA CAC TCC CTA CT |
| 5g09580_P1 | *RodA* | CGT GCT CCA TAT TTC CGA TT (20 bp) |
| 5g09580_P2 | *RodA* | TAG TTC TGT TAC CGA GCC GGT GGA ATG TGG TAT GCA AGG A (40 bp) |
| 5g09580_P3 | *RodA* | GCT CTG AAC GAT ATG CTC CCC CAG CAT TTG GAG ATT CGA T (40 bp) |
| 5g09580_P4 | *RodA* | TTT GCG AGT AAC GGT GTT TG (20 bp) |
| 5g09580_P5 | *RodA* | TTG CAG ACC AAC AAC AGA GC (20 bp) |
| 5g09580_P6 | *RodA* | GGT TTG GGG GTC TAC GAT CT (20 bp) |
| hph_F |  | CCG GCT CGG TAA CAG AAC TAA CGG CGT AAC CAA AAG TCA C (40 bp) |
| hph_R |  | GGG AGC ATA TCG TTC AGA GCT CTT GAC GAC CGT TGA TCT G (40 bp) |
